# Supplementary material for: Identification of Flavanone 3-Hydroxylase Gene Family in Strawberry and Expression Analysis of Fruit at Different Coloring Stages
Source: Int J Mol Sci. 2023 Nov 27;24(23):16807. doi: 10.3390/ijms242316807 (PMC10706444; doi:10.3390/ijms242316807)
Supplement: Supplementary file 1 [file ijms-24-16807-s001.zip › ijms-2679279-SI.pdf]

Supplementary Table S1. The physicochemical properties of *FvF3H* in strawberry

Gene name	Accession number	Amino acid numbers(aa)	Molecular Weight	Theoretical pI	Instability Index	Aliphatic Index	Grand Average of Hydropathicity
FvF3H1	*FvH4_1g00520.t1*	311	35156.51	5.58	29.38	77.11	-0.421
FvF3H2	*FvH4_1g02111.t1*	359	40328.1	5.97	43.24	87.33	-0.324
FvF3H3	*FvH4_1g02112.t1*	339	38426	5.37	43.18	87.61	-0.291
FvF3H4	*FvH4_1g02120.t1*	339	38701.36	5.24	42.98	91.36	-0.345
FvF3H5	*FvH4_1g02121.t1*	354	40771.94	5.33	49.93	86.16	-0.391
FvF3H6	*FvH4_1g02150.t1*	355	40623.73	4.83	48.28	94.39	-0.275
FvF3H7	*FvH4_1g02151.t1*	354	40680.58	4.93	48.38	91.69	-0.317
FvF3H8	*FvH4_1g02152.t1*	354	40680.58	4.78	44.55	90.79	-0.259
FvF3H9	*FvH4_1g02160.t1*	265	31220.56	4.95	51.17	75.02	-0.513
FvF3H10	*FvH4_1g04200.t1*	355	39947.51	6.19	39.02	85.1	-0.253
FvF3H11	*FvH4_1g05410.t1*	372	41691.52	6.1	37.12	86.24	-0.289
FvF3H12	*FvH4_1g06010.t4*	281	32158.62	5.49	38.42	82.56	-0.443
FvF3H13	*FvH4_1g07181.t1*	261	29506.24	5.29	37.02	76.13	-0.556
FvF3H14	*FvH4_1g07190.t1*	307	35042.92	5.61	33.66	75.31	-0.535
FvF3H15	*FvH4_1g09830.t1*	297	33103.08	5.31	38.78	93.84	-0.128
FvF3H16	*FvH4_1g11810.t1*	364	41080.8	5.52	41.11	80.88	-0.476
FvF3H17	*FvH4_1g16460.t3*	239	26766.56	5.18	20.15	78.28	-0.307
FvF3H18	*FvH4_1g26760.t1*	345	37962.26	5.93	47.89	83.36	-0.237
FvF3H19	*FvH4_2g03190.t1*	239	26385.11	4.93	42.11	94.64	-0.226
FvF3H20	*FvH4_2g03200.t1*	608	67909.56	5.15	40.97	92.83	-0.208

FvF3H21	*FvH4_2g03210.t1*	605	68294.08	5.22	38.91	92.64	-0.238
FvF3H22	*FvH4_2g03220.t1*	187	20650.66	5.91	35.96	90.64	-0.26
FvF3H23	*FvH4_2g07071.t1*	326	37682.73	5.76	34.68	77.73	-0.695
FvF3H24	*FvH4_2g07080.t2*	351	40619.94	5.68	39.69	75.21	-0.65
FvF3H25	*FvH4_2g07081.t1*	353	40681.17	5.33	38.28	80	-0.594
FvF3H26	*FvH4_2g07082.t1*	351	40909.63	5.7	32.52	81.57	-0.504
FvF3H27	*FvH4_2g15310.t1*	348	39232.33	5.44	48.8	81.15	-0.348
FvF3H28	*FvH4_2g15312.t1*	151	17122.77	9.65	41.77	75.5	-0.481
FvF3H29	*FvH4_2g21630.t1*	344	39115.7	5.77	33.81	86.57	-0.289
FvF3H30	*FvH4_2g21690.t3*	342	38646.97	5.57	42.69	84.55	-0.324
FvF3H31	*FvH4_2g21772.t1*	349	39665.59	5.58	41.24	99.63	-0.133
FvF3H32	*FvH4_2g21773.t1*	337	38199.71	5.47	42.31	99.14	-0.158
FvF3H33	*FvH4_2g21774.t1*	342	38275.81	6	45.18	89.74	-0.278
FvF3H34	*FvH4_2g21830.t1*	361	40802.4	5.12	51.81	86.3	-0.34
FvF3H35	*FvH4_2g21870.t1*	305	34043.06	5.17	42.28	76.36	-0.452
FvF3H36	*FvH4_2g21890.t1*	365	41210.87	6.51	30.86	88.38	-0.354
FvF3H37	*FvH4_3g21910.t1*	329	37755.26	6.64	43.42	82.61	-0.316
FvF3H38	*FvH4_2g26440.t1*	335	38227.56	5.41	38.33	84.36	-0.529
FvF3H39	*FvH4_2g26480.t1*	289	32741.61	5.17	50.3	90.35	-0.329
FvF3H40	*FvH4_2g27140.t1*	342	38594.17	5.55	33.99	85.79	-0.205
FvF3H41	*FvH4_2g29950.t1*	318	36117.15	5.42	39.43	79.12	-0.383
FvF3H42	*FvH4_2g29991.t1*	326	37202.67	4.95	45.22	79.17	-0.256
FvF3H43	*FvH4_2g30010.t1*	336	37704.27	6.79	36.84	90.77	-0.212
FvF3H44	*FvH4_2g30020.t1*	341	38444	5.47	36.9	92.61	-0.167
FvF3H45	*FvH4_2g30021.t1*	341	37915.37	5.21	36.32	92.61	-0.123
FvF3H46	*FvH4_2g30040.t1*	353	39308.95	6.19	44.28	87.85	-0.122

FvF3H47	*FvH4_2g35050.t1*	375	42527.54	7.63	28.65	79.52	-0.308
FvF3H48	*FvH4_3g01280.t1*	312	35555.86	5.11	38.4	80	-0.385
FvF3H49	*FvH4_3g02670.t1*	350	39812.94	5.89	52.31	68.8	-0.486
FvF3H50	*FvH4_3g05530.t1*	336	37704.19	6.03	40.76	83.01	-0.213
FvF3H51	*FvH4_3g06511.t3*	331	37576.47	5.48	45.25	91.04	-0.247
FvF3H52	*FvH4_3g06513.t2*	180	20261.2	5.64	37.16	87.72	-0.349
FvF3H53	*FvH4_3g07861.t1*	284	31701.18	5.11	41.49	87.5	-0.285
FvF3H54	*FvH4_3g12330.t1*	299	34345.34	5.15	41.1	73.65	-0.4
FvF3H55	*FvH4_3g12340.t1*	316	35973.02	4.95	40.65	79.3	-0.283
FvF3H56	*FvH4_3g15580.t1*	366	40353.21	5.58	33.72	89.7	-0.211
FvF3H57	*FvH4_3g16760.t1*	337	37728.85	6.1	45.49	82.14	-0.291
FvF3H58	*FvH4_3g17470.t1*	375	42592.59	5.15	39.08	87.36	-0.358
FvF3H59	*FvH4_3g21910.t1*	329	37755.26	6.64	43.42	82.61	-0.316
FvF3H60	*FvH4_3g23800.t1*	299	34389.24	5.49	34.88	75.28	-0.428
FvF3H61	*FvH4_3g28210.t1*	316	36026.81	5.2	34.59	75.85	-0.433
FvF3H62	*FvH4_3g31880.t3*	292	32981.03	6.23	44.71	97.77	-0.157
FvF3H63	*FvH4_3g36530.t1*	344	38518.9	5.43	31.18	74.77	-0.309
FvF3H64	*FvH4_3g38920.t1*	334	37594.87	6	32.93	87.75	-0.3
FvF3H65	*FvH4_3g45310.t1*	364	40816.76	5.91	48.55	87.25	-0.273
FvF3H66	*FvH4_4g03240.t1*	347	38599.8	5.09	39.38	94.7	-0.228
FvF3H67	*FvH4_4g03241.t1*	355	40483.83	5.4	40.43	79.58	-0.467
FvF3H68	*FvH4_4g03250.t1*	362	40794.32	5.14	34.07	88.51	-0.332
FvF3H69	*FvH4_4g03251.t1*	361	40854.47	5.77	33.68	86.34	-0.365
FvF3H70	*FvH4_4g03260.t1*	369	41479.06	5.32	42.24	83.44	-0.363
FvF3H71	*FvH4_4g03301.t1*	288	32421.81	5.81	38.61	79.34	-0.444
FvF3H72	*FvH4_4g03303.t1*	360	40671.12	5.52	32.83	84.97	-0.396

FvF3H73	*FvH4_4g03304.t1*	380	43013.35	6.61	34.92	89.95	-0.334
FvF3H74	*FvH4_4g03305.t1*	366	40798.57	5.41	32	93.72	-0.165
FvF3H75	*FvH4_4g03323.t1*	295	33764.57	5.78	47.38	84.74	-0.331
FvF3H76	*FvH4_4g14690.t1*	256	28359.51	4.94	29.05	95.55	-0.035
FvF3H77	*FvH4_4g17720.t1*	365	40986	7.62	37.52	87.01	-0.335
FvF3H78	*FvH4_4g20730.t1*	319	35014.95	5.9	40.87	82.48	-0.159
FvF3H79	*FvH4_4g25590.t1*	339	39053.48	5.92	32.91	87.4	-0.417
FvF3H80	*FvH4_4g33740.t1*	362	39661.35	5.56	36.92	89.09	-0.194
FvF3H81	*FvH4_4g36510.t2*	259	28983.96	5.48	51.48	86.15	-0.269
FvF3H82	*FvH4_5g01170.t1*	383	42861.12	5.48	46.41	92.17	-0.33
FvF3H83	*FvH4_5g10430.t1*	345	39074.61	5.74	41.35	88.99	-0.323
FvF3H84	*FvH4_5g19290.t1*	312	35234.04	4.77	40.15	93.01	-0.186
FvF3H85	*FvH4_5g19300.t1*	483	54666	8.96	69.78	68.22	-0.689
FvF3H86	*FvH4_5g19310.t2*	302	33932.47	5.19	43.63	78.85	-0.414
FvF3H87	*FvH4_5g19321.t1*	285	31848.09	5.36	38.42	85.62	-0.358
FvF3H88	*FvH4_5g19970.t1*	358	40517.57	6.13	43.3	87.4	-0.278
FvF3H89	*FvH4_5g20420.t1*	312	34710.37	5.59	31.19	88.43	-0.215
FvF3H90	*FvH4_5g20421.t1*	365	40886.68	5.97	36.53	81.78	-0.306
FvF3H91	*FvH4_5g20430.t1*	355	39778.23	5.75	35.86	82.2	-0.304
FvF3H92	*FvH4_5g20431.t1*	357	40197.75	5.6	36.63	83.36	-0.316
FvF3H93	*FvH4_5g20432.t5*	357	40197.75	5.49	34.82	82.55	-0.318
FvF3H94	*FvH4_5g21360.t1*	542	61435.81	5.35	42.5	81.64	-0.402
FvF3H95	*FvH4_5g26041.t1*	171	19022.78	5.55	44.47	91.75	-0.1
FvF3H96	*FvH4_5g33280.t1*	348	39301.64	5.13	36.86	80.78	-0.341
FvF3H97	*FvH4_5g35660.t1*	369	41255.88	5.85	32.74	85.91	-0.321
FvF3H98	*FvH4_5g38040.t1*	357	39577.12	5.25	39.83	84.76	-0.245

FvF3H99	*FvH4_5g38050.t1*	355	39316.79	5.25	47.02	85.24	-0.225
FvF3H100	*FvH4_6g13281.t1*	166	18588.46	7.79	38.39	87.47	-0.356
FvF3H101	*FvH4_6g13290.t1*	364	40867.68	5.52	47.44	89.45	-0.352
FvF3H102	*FvH4_6g13300.t1*	365	41496.31	5.3	47.68	92.03	-0.318
FvF3H103	*FvH4_6g13322.t1*	355	40120.96	5.74	35.31	91.94	-0.35
FvF3H104	*FvH4_6g17160.t1*	477	54548.55	6.35	54.04	86.79	-0.23
FvF3H105	*FvH4_6g19280.t1*	369	41416.18	5.43	37.59	88.34	-0.234
FvF3H106	*FvH4_6g19290.t1*	208	23069.47	5.17	33.22	98.41	-0.057
FvF3H107	*FvH4_6g27770.t1*	340	38439.03	5.32	46.09	86.24	-0.363
FvF3H108	*FvH4_6g28170.t1*	366	41096.17	5.45	41.19	91.28	-0.283
FvF3H109	*FvH4_6g30750.t1*	313	35890.93	5.4	37.47	76.9	-0.36
FvF3H110	*FvH4_6g30760.t1*	316	36040.06	5.72	33.42	82.34	-0.384
FvF3H111	*FvH4_6g30780.t1*	358	40063.75	6.62	48.41	88.77	-0.227
FvF3H112	*FvH4_6g39151.t1*	348	38753.67	5.49	46.41	85.46	-0.373
FvF3H113	*FvH4_6g42090.t1*	320	36233.31	5.08	31.46	80.75	-0.409
FvF3H114	*FvH4_6g42700.t1*	316	36088.89	5.19	36.43	74.62	-0.438
FvF3H115	*FvH4_7g06780.t1*	384	43322.95	5.47	47.45	80.16	-0.393
FvF3H116	*FvH4_7g10320.t1*	208	23307.68	5.08	36.89	93.17	-0.275
FvF3H117	*FvH4_7g10930.t1*	167	19272.68	5.97	30.02	80.54	-0.503
FvF3H118	*FvH4_7g12600.t1*	379	42918.84	5.34	28.38	78.42	-0.268
FvF3H119	*FvH4_7g12610.t2*	348	39185.53	5.82	32.93	79.2	-0.351
FvF3H120	*FvH4_7g14240.t1*	339	38518.66	5.92	44.51	75.28	-0.384
FvF3H121	*FvH4_7g15780.t1*	316	35913.63	5.72	40.39	69.37	-0.491
FvF3H122	*FvH4_7g17840.t1*	345	38965.13	5.79	39.05	86.72	-0.362
FvF3H123	*FvH4_7g28670.t1*	373	42457.11	5.52	35.14	70.05	-0.449
FvF3H124	*FvH4_7g30770.t2*	371	41776.35	5.23	38.4	77.45	-0.381

FvF3H125	*FvH4_7g31470.t1*	401	45397.51	6.44	35.56	90.42	-0.259
FvF3H126	*FvH4_7g32980.t1*	338	38619.84	5.84	44.46	83.58	-0.436

Supplementary Table S2. The secondary structure prediction and subcellular location prediction of *FvF3H* proteins

name	Alpha helix	Random coil	Extended strand	Subcellular location
FvF3H1	26.37%	53.05%	20.58%	cytoplasm nucleus cytoskeleton chloroplast extracellular
FvF3H2	31.75%	44.85%	23.40%	cytoplasm chloroplast nucleus
FvF3H3	38.35%	44.25%	17.40%	nucleus cytoskeleton cytoplasm extracellular
FvF3H4	38.35%	44.25%	17.40%	nucleus cytoplasm chloroplast extracelular cytoskeleon
FvF3H5	36.72%	48.02%	15.25%	cytoplasm chloroplast cytoskeleton nucleus
FvF3H6	36.72%	48.02%	15.25%	cytoplasm chloroplast nucleus cytoskeleton
FvF3H7	42.66%	44.35%	12.99%	cytoplasm cytoskeleton nucleus
FvF3H8	33.99%	47.88%	18.13%	cytoplasm cytoskeleton chloroplast nucleus Golgi apparatus
FvF3H9	33.96%	50.57%	15.47%	cytoplasm nucleus
FvF3H10	33.96%	50.57%	15.47%	chloroplast nucleus cytoplasm extracellular
FvF3H11	34.68%	49.19%	16.13%	cytoskeleton nucleus
FvF3H12	34.88%	50.53%	14.59%	cytoplasm nucleus mitochondrion chloroplast cytoskeleton
FvF3H13	33.44%	48.20%	18.36%	nucleus cytoplasm plasma membrane cytoskeleton
FvF3H14	32.57%	51.14%	16.29%	cytoplasm nucleus extracellular cytoskeleton Golgi apparatus
FvF3H15	38.72%	43.77%	17.51%	nucleus cytoskeleton cytoplasm endoplasmic reticulum vacular membrane
FvF3H16	30.77%	48.35%	20.88%	nucleus cytoplasm chloroplast plasma membrane mitochondrion plasma membrane endoplasmic reticulum cytoskeleton
FvF3H17	37.66%	49.79%	12.55%	cytoplasm mitochondrion plasma membrane Golgi apparatus cytoskeleton nucleus
FvF3H18	28.70%	57.68%	13.62%	nucleus cytoplasm chloroplast mitochondrion plasma membrane extracellular
FvF3H19	43.10%	46.44%	10.46%	cytoplasm cytoskeleton plasma membrane mitochondrion Endoplasmic reticulum peroxisome
FvF3H20	45.39%	40.62%	13.98%	cytoplasm nucleus chloroplast Golgi apparatus
FvF3H21	48.10%	38.35%	13.55%	cytoplasm chloroplast nucleus peroxisome Golgi apparatus

FvF3H22	15.95%	15.95%	15.95%	cytoplasm cytoskeleton extracellular Golgi apparatus
FvF3H23	29.45%	54.60%	15.95%	cytoskeleton cytoplasm endoplasmic reticulum vacular membrane nucleus
FvF3H24	30.20%	55.27%	14.53%	cytoplasm nucleus cytoskeleton
FvF3H25	28.05%	51.84%	20.11%	cytoplasm nucleus cytoskeleton chloroplast endoplasmic reticulum vacular membrane
FvF3H26	33.90%	45.87%	20.23%	nucleus cytoplasm chloroplast cytoskeleton
FvF3H27	24.71%	53.16%	22.13%	cytoplasm nucleus chloroplast plasma membrane
FvF3H28	33.11%	49.67%	17.22%	cytoplasm nucleus
FvF3H29	32.15%	45.50%	22.34%	cytoplasm nucleus cytoskeleton
FvF3H30	34.21%	48.12%	17.67%	cytoplasm nucleus chloroplast extracellular
FvF3H31	30.66%	47.28%	22.06%	cytoplasm nucleus chloroplast extracellular cytoskeleton
FvF3H32	32.94%	45.99%	21.07%	chloroplast mitochondrion peroxisome cytoplasm nucleus
FvF3H33	30.12%	51.17%	18.71%	cytoplasm nucleus peroxisome Golgi apparatus
FvF3H34	35.94%	45.20%	18.86%	cytoplasm nucleus
FvF3H35	29.51%	53.44%	17.05%	nucleus cytoplasm chloroplast mitochondrion plasma membrane
FvF3H36	38.36%	48.22%	13.42%	cytoplasm nucleus chloroplast vacular membrane
FvF3H37	36.17%	44.68%	19.15%	nucleus cytoplasm extracellular cytoskeleton
FvF3H38	29.25%	52.54%	18.21%	cytoplasm nucleus cytoskeleton plasm membrane chloroplast Golgi apparatus
FvF3H39	39.45%	42.56%	17.99%	cytoplasm Endoplasmic reticulum chloroplast nucleus mitochondrion
FvF3H40	29.53%	52.05%	18.42%	nucleus cytoplasm cytoskeleton plasma membrane
FvF3H41	41.51%	45.60%	12.89%	nucleus cytoplasm chloroplast cytoskeleton
FvF3H42	36.81%	49.08%	14.11%	cytoplasm nucleus chloroplast cytoskeleton extracellular
FvF3H43	31.55%	52.08%	16.37%	cytoplasm nucleus cytoskeleton extracellular endoplasmic reticulum vacular membrane
FvF3H44	36.66%	47.80%	15.54%	cytoskeleton nucleus cytoplasm
FvF3H45	39.30%	47.21%	13.49%	cytoplasm nucleus peroxisome
FvF3H46	37.39%	48.16%	14.45%	cytoplasm nucleus chloroplast
FvF3H47	39.20%	48.27%	12.53%	chloroplast mitochondrion cytoplasm plasma membrane

FvF3H48	39.10%	45.51%	15.38%	cytoplasm nucleus chloroplast cytoskeleton
FvF3H49	35.71%	50.00%	14.29%	nucleus chloroplast extracellular vacular membrane
FvF3H50	31.55%	46.13%	22.32%	nucleus cytoplasm chloroplast Golgi apparatus
FvF3H51	36.81%	47.22%	15.97%	cytoplasm Endoplasmic reticulum nucleus cytoskeleyon
FvF3H52	27.78%	50.00%	22.22%	cytoplasm nucleus
FvF3H53	41.90%	46.48%	11.62%	nucleus cytoplasm extracellular cytoskeleton Golgi apparatus Endoplasmic reticulum vacular membrane
FvF3H54	40.13%	46.82%	13.04%	nucleus chloroplast cytoskeleton cytoskeleton
FvF3H55	42.41%	46.20%	11.39%	nucleus cytoplasm cytoskeleton
FvF3H56	32.51%	48.63%	18.85%	cytiplasm extracellular cytoskeleton chloroplast
FvF3H57	29.08%	48.66%	22.26%	chloroplast mitochondrion nucleus mitochondrion cytoplasm plasma membrane
FvF3H58	39.47%	46.93%	13.60%	cytoplasm nucleus extracellular Golgi apparatus
FvF3H59	36.17%	44.68%	19.15%	nucleus cytoplasm extracellular cytoskeleton
FvF3H60	38.13%	46.49%	15.38%	nucleus chloroplast extracellular
FvF3H61	31.96%	52.22%	15.82%	cytoplasm nucleus extracellular vacular membrane cytoskeleton
FvF3H62	43.15%	36.64%	20.21%	cytoplasm chloroplast nucleus peroxisome cytoskeleton
FvF3H63	23.55%	57.56%	18.90%	nucleus cytoplasm chloroplast vacular membrane
FvF3H64	21.56%	59.58%	18.86%	nucleus cytoplasm chloroplast mitochondrion plasma membrane cytoskeleton
FvF3H65	36.54%	47.25%	16.21%	peroxisome cytoplasm nucleus extracellular
FvF3H66	32.28%	52.16%	15.56%	cytoplasm nucleus extracellular cytoskeleton
FvF3H67	29.58%	54.93%	15.49%	nucleus cytoplasm peroxisome extracellular cytoskeleton
FvF3H68	30.11%	56.63%	13.26%	cytoplasm mitochondrion chloroplast nucleus endoplasmic reticulum peroxisome
FvF3H69	32.13%	55.40%	12.47%	cytoplasm nucleus chloroplast mitochondrion plasma membrane endoplasmic reticulum
FvF3H70	26.56%	56.64%	16.80%	cytoplasm nucleus peroxisome
FvF3H71	28.42%	57.92%	13.66%	cytoplasm nucleus cytoskeleton chloroplast peroxisome
FvF3H72	34.44%	49.44%	16.11%	cytoplasm nucleus chloroplast extracellular cytoskeleton

FvF3H73	38.16%	43.68%	18.16%	chloroplast mitochondrion cytoplasm nucleus vacular membrane
FvF3H74	38.52%	48.36%	13.11%	cytoplasm nucleus peroxisome
FvF3H75	40.17%	45.98%	13.85%	cytoplasm nucleus cytoskeleton
FvF3H76	43.75%	41.41%	14.84%	cytoplasm chloroplast nucleus
FvF3H77	32.60%	54.25%	13.15%	nucleus cytoplasm chloroplast cytoskeleton
FvF3H78	29.78%	52.04%	18.18%	cytoplasm nucleus extracellular chloroplast cytoskeleton Golgi apparatus
FvF3H79	33.33%	48.67%	17.99%	cytoplasm chloroplast mitochondrion plasma membrane nucleus cytoskeleton
FvF3H80	26.52%	53.31%	20.17%	cytoplasm cytoskeleton nucleus extracellular
FvF3H81	36.72%	51.34%	11.94%	nucleus cytoplasm chloroplast extracellular
FvF3H82	39.95%	45.69%	14.36%	chloroplast cytoplasm cytoskeleton nucleus mitochondrion Golgi apparatus
FvF3H83	39.13%	47.25%	13.62%	cytoplasm nucleus cytoskeleton plasma membrane extracellular
FvF3H84	39.42%	50.64%	9.94%	cytoplasm mitochondrion Chloroplast plasma membrane Endoplasmic reticulum
FvF3H85	26.09%	62.11%	11.80%	nucleus chloroplast cytoplasm cytoskeleton
FvF3H86	32.99%	54.17%	12.85%	cytoplasm nucleus mitochondrion plasma membrane peroxisome
FvF3H87	33.88%	49.59%	16.53%	cytoplasm chloroplast nucleus cytoskeleton endoplasmic reticulum vacular membrane
FvF3H88	36.03%	49.16%	14.80%	cytoplasm nucleus cytoskeleton chloroplast
FvF3H89	32.69%	44.23%	23.08%	cytoplasm nucleus chloroplast
FvF3H90	33.15%	47.95%	18.90%	cytoskeleton cytoplasm nucleus
FvF3H91	40.56%	43.94%	15.49%	cytoskeleton nucleus chloroplast cytoplasm plasma membrane
FvF3H92	42.58%	41.46%	15.97%	cytoskeleton
FvF3H93	34.73%	48.46%	16.81%	cytoskeleton cytoplasm
FvF3H94	42.80%	43.91%	13.28%	cytoplasm vacular membrane endoplasmic reticulum Golgi apparatus chloroplast plasma membrane
FvF3H95	32.75%	54.97%	12.28%	cytoplasm nucleus extracellular chloroplast mitochondrion cytoskeleton Golgi apparatus
FvF3H96	38.79%	45.11%	16.09%	cytoskeleton cytoplasm nucleus plasma membrane
FvF3H97	35.50%	50.68%	13.82%	cytoplasm nucleus extracellular peroxisome

FvF3H98	37.25%	47.90%	14.85%	cytoskeleton cytoplasm nucleus
FvF3H99	38.03%	47.61%	14.37%	cytoplasm cytoskeleton nucleus chloroplast vacular membrane
FvF3H100	18.07%	55.42%	26.51%	cytoplasm extracellular nucleus cytoskeleton Golgi apparatus
FvF3H101	30.30%	54.55%	15.15%	cytoplasm cytoskeleton nucleus chloroplast
FvF3H102	33.97%	49.32%	16.71%	cytoplasm extracellular cytoskeleton
FvF3H103	31.83%	44.79%	23.38%	cytoplasm chloroplast nucleus
FvF3H104	35.22%	48.85%	15.93%	chloroplast vacular membrane nucleus mitochondrion extracellular Golgi apparatus
FvF3H105	33.45%	49.31%	17.24%	cytoplasm nucleus mitochondrion peroxisome
FvF3H106	37.98%	43.27%	18.75%	cytoplasm extracellular cytoskeleton Golgi apparatus
FvF3H107	28.53%	49.12%	22.35%	cytoplasm nucleus chloroplast nucleus peroxisome Golgi apparatus
FvF3H108	32.79%	48.09%	19.13%	cytoplasm cytoskeleton chloroplast nucleus peroxisome
FvF3H109	32.91%	51.44%	15.65%	nucleus cytoplasm extracellular chloroplast cytoskeleton
FvF3H110	34.49%	51.27%	14.24%	cytoplasm nucleus extracellular peroxisome cytoskeleton
FvF3H111	29.05%	53.91%	17.04%	nucleus cytoplasm plasma membrane Golgi apparatus plasma membrane mitochondrion peroxisome cytoskeleton
FvF3H112	26.44%	53.16%	20.40%	cytoplasm nucleus cytoskeleton
FvF3H113	38.12%	45.00%	16.88%	cytoplasm cytoskeleton chloroplast nucleus
FvF3H114	31.65%	52.85%	15.51%	nucleus cytoplasm chloroplast extracellular vacular membrane
FvF3H115	27.34%	56.51%	16.15%	nucleus chloroplast cytoplasm extracellular vacular membrane
FvF3H116	23.08%	52.88%	24.04%	nucleus mitochondrion cytoplasm extracellular cytoskeleton
FvF3H117	32.93%	48.50%	18.56%	cytoskeleton nucleus cytoplasm extracellular Golgi apparatus
FvF3H118	36.41%	50.66%	12.93%	cytoplasm nucleus peroxisome mitochondrion extracellular vacular membrane
FvF3H119	31.73%	49.60%	18.67%	nucleus cytoplasm cytoskeleton chloroplast
FvF3H120	30.38%	48.97%	20.65%	nucleus cytoplasm chloroplast extracellular vacular membrane
FvF3H121	25.63%	55.70%	18.67%	nucleus cytoplasm chloroplast extracellular vacular membrane
FvF3H122	24.35%	54.49%	21.16%	cytoplasm chloroplast cytoskeleton plasma membrane nucleus

FvF3H123	38.07%	46.38%	15.55%	nucleus cytoskeleton extracellular vacular membrane
FvF3H124	42.61%	46.67%	10.72%	cytoplasm nucleus chloroplast mitochondrion extracellular vacular membrane
FvF3H125	42.89%	43.64%	13.47%	mitochondrion cytoplasm mitochondrion nucleus chloroplast plasma membrane Golgi apparatus
FvF3H126	34.02%	44.97%	21.01%	peroxisome chloroplast nucleus mitochondrion cytoplasm

Supplementary Table S3. *FvF3H* gene codon preference parameter

name	T3s	C3s	A3s	G3s	CAI	CBI	Fop	Nc	GC3s	GC
FvF3H1	0.3891	0.3463	0.274	0.2969	0.263	0.008	0.432	56	0.334	0.419
FvF3H2	0.4245	0.205	0.3755	0.2548	0.186	-0.112	0.345	47.64	0.357	0.426
FvF3H3	0.4038	0.2154	0.3977	0.2627	0.187	-0.14	0.331	49.28	0.358	0.414
FvF3H4	0.419	0.2253	0.3969	0.251	0.209	-0.064	0.373	51.03	0.36	0.417
FvF3H5	0.366	0.2453	0.4045	0.2851	0.196	-0.058	0.375	55.82	0.361	0.428
FvF3H6	0.4296	0.2222	0.3603	0.2782	0.188	-0.073	0.362	50.34	0.362	0.421
FvF3H7	0.386	0.2316	0.3985	0.2794	0.182	-0.088	0.356	52.19	0.362	0.408
FvF3H8	0.3963	0.2222	0.4231	0.2692	0.182	-0.096	0.353	49.14	0.37	0.417
FvF3H9	0.3618	0.2663	0.4043	0.3118	0.189	-0.095	0.366	52.2	0.373	0.425
FvF3H10	0.2577	0.378	0.3008	0.3165	0.213	0.052	0.442	56.23	0.375	0.424
FvF3H11	0.2727	0.3434	0.3223	0.3548	0.198	-0.064	0.382	59.3	0.376	0.423
FvF3H12	0.4253	0.2353	0.3532	0.2857	0.203	-0.039	0.391	55.9	0.38	0.424
FvF3H13	0.3906	0.3262	0.3226	0.2864	0.255	0.028	0.446	60	0.38	0.453
FvF3H14	0.3463	0.3723	0.3239	0.3073	0.241	-0.004	0.429	59.41	0.381	0.416
FvF3H15	0.3853	0.2641	0.3	0.3175	0.222	-0.01	0.406	49.44	0.382	0.417
FvF3H16	0.25	0.4118	0.1893	0.4466	0.248	0.06	0.454	52.95	0.383	0.443
FvF3H17	0.4149	0.2128	0.4048	0.2763	0.2	-0.084	0.377	51.99	0.385	0.429
FvF3H18	0.3275	0.331	0.2969	0.2929	0.208	-0.026	0.399	58	0.385	0.413
FvF3H19	0.4316	0.2842	0.2889	0.2654	0.205	-0.043	0.385	58.94	0.391	0.439
FvF3H20	0.4141	0.2381	0.3094	0.3175	0.195	-0.063	0.376	53.7	0.391	0.449
FvF3H21	0.4198	0.2363	0.3009	0.3317	0.197	-0.056	0.38	53.35	0.392	0.431
FvF3H22	0.4065	0.2645	0.2609	0.344	0.195	-0.079	0.361	59.57	0.394	0.432

FvF3H23	0.3936	0.2771	0.3291	0.3077	0.199	-0.019	0.403	48.37	0.394	0.432
FvF3H24	0.3596	0.3034	0.3256	0.3237	0.192	-0.078	0.372	52.42	0.397	0.427
FvF3H25	0.3902	0.2917	0.3233	0.3012	0.206	-0.06	0.378	54.81	0.399	0.429
FvF3H26	0.3759	0.2895	0.3482	0.319	0.196	-0.086	0.362	51.1	0.399	0.441
FvF3H27	0.4078	0.305	0.3045	0.2844	0.216	-0.048	0.395	59.54	0.4	0.423
FvF3H28	0.3361	0.3361	0.3636	0.25	0.188	-0.038	0.4	57.1	0.4	0.417
FvF3H29	0.3883	0.2747	0.3696	0.2643	0.181	-0.114	0.348	54.7	0.401	0.432
FvF3H30	0.3684	0.2727	0.2979	0.3352	0.219	0.036	0.438	58.57	0.402	0.435
FvF3H31	0.4038	0.2346	0.3802	0.2785	0.186	-0.07	0.364	49.02	0.405	0.433
FvF3H32	0.4022	0.2362	0.3825	0.2731	0.183	-0.078	0.36	49.03	0.41	0.433
FvF3H33	0.386	0.3051	0.3098	0.2689	0.229	0.003	0.411	55.03	0.41	0.444
FvF3H34	0.3241	0.2828	0.3811	0.298	0.194	-0.029	0.397	52.38	0.411	0.438
FvF3H35	0.3516	0.2912	0.358	0.2532	0.218	0.019	0.433	61	0.414	0.426
FvF3H36	0.3367	0.3299	0.2989	0.3125	0.232	0.021	0.426	58.7	0.414	0.426
FvF3H37	0.3891	0.2802	0.3745	0.276	0.207	-0.041	0.393	56.94	0.414	0.442
FvF3H38	0.3092	0.4056	0.2351	0.3739	0.246	-0.014	0.409	54.7	0.417	0.423
FvF3H39	0.327	0.3081	0.2589	0.4	0.23	-0.021	0.395	55.14	0.419	0.427
FvF3H40	0.4396	0.2857	0.264	0.2788	0.217	-0.053	0.382	56.33	0.42	0.469
FvF3H41	0.4298	0.2314	0.3249	0.3018	0.197	-0.082	0.375	58.85	0.421	0.443
FvF3H42	0.2803	0.4017	0.2308	0.3839	0.257	0.044	0.442	54.75	0.424	0.433
FvF3H43	0.4228	0.2904	0.2451	0.2906	0.205	-0.01	0.401	53.65	0.425	0.432
FvF3H44	0.3755	0.2996	0.304	0.2902	0.216	-0.064	0.372	58.28	0.426	0.444
FvF3H45	0.3835	0.3047	0.2741	0.2731	0.235	0.006	0.412	59.54	0.426	0.439
FvF3H46	0.3945	0.3495	0.229	0.2614	0.237	0.033	0.435	56.52	0.427	0.436
FvF3H47	0.3828	0.3333	0.2667	0.3031	0.22	-0.015	0.413	57.22	0.431	0.466
FvF3H48	0.2655	0.4381	0.2694	0.3689	0.261	0.007	0.425	53.2	0.432	0.452

FvF3H49	0.347	0.3134	0.304	0.3502	0.228	-0.021	0.42	59.2	0.432	0.452
FvF3H50	0.2937	0.3457	0.3052	0.3275	0.204	0.007	0.421	57.64	0.433	0.466
FvF3H51	0.375	0.2419	0.3413	0.3234	0.187	-0.078	0.356	56.84	0.434	0.432
FvF3H52	0.375	0.2419	0.3413	0.3234	0.187	-0.078	0.356	56.84	0.438	0.445
FvF3H53	0.3586	0.2759	0.2836	0.3583	0.168	-0.108	0.347	57.01	0.439	0.438
FvF3H54	0.4178	0.2933	0.267	0.3016	0.206	-0.042	0.386	55.85	0.44	0.45
FvF3H55	0.376	0.2727	0.4	0.2598	0.216	-0.027	0.41	55.04	0.442	0.447
FvF3H56	0.3655	0.2517	0.3125	0.3413	0.216	-0.034	0.394	55.02	0.443	0.452
FvF3H57	0.3686	0.2993	0.3401	0.2655	0.199	0.008	0.425	47.97	0.444	0.464
FvF3H58	0.4113	0.2908	0.3404	0.2548	0.215	-0.021	0.404	57.49	0.444	0.433
FvF3H59	0.3891	0.2802	0.3745	0.276	0.207	-0.041	0.393	56.94	0.446	0.449
FvF3H60	0.4053	0.2599	0.3692	0.2736	0.231	-0.039	0.4	52.86	0.448	0.462
FvF3H61	0.2828	0.4631	0.2588	0.2991	0.244	0.035	0.443	58.49	0.448	0.44
FvF3H62	0.3448	0.2974	0.2864	0.3483	0.19	-0.037	0.387	59.11	0.448	0.442
FvF3H63	0.339	0.2877	0.3007	0.3506	0.191	-0.015	0.401	58.41	0.448	0.457
FvF3H64	0.386	0.3235	0.2869	0.2641	0.205	0	0.407	52.08	0.449	0.444
FvF3H65	0.3604	0.2968	0.3407	0.2749	0.18	-0.116	0.348	56.76	0.45	0.451
FvF3H66	0.4055	0.2268	0.377	0.2684	0.177	-0.105	0.352	53.82	0.451	0.459
FvF3H67	0.4488	0.2261	0.3452	0.2863	0.204	-0.124	0.345	55.68	0.452	0.443
FvF3H68	0.4521	0.2226	0.3422	0.2713	0.211	-0.054	0.379	52.33	0.452	0.474
FvF3H69	0.4539	0.2287	0.3561	0.243	0.218	-0.067	0.372	55.81	0.453	0.453
FvF3H70	0.4203	0.2542	0.3371	0.2713	0.205	-0.065	0.379	50.69	0.454	0.476
FvF3H71	0.4509	0.2589	0.3237	0.2618	0.229	-0.034	0.402	55.27	0.454	0.464
FvF3H72	0.2889	0.294	0.3284	0.3414	0.174	-0.076	0.366	55.74	0.454	0.453
FvF3H73	0.3787	0.2924	0.2817	0.3269	0.195	-0.049	0.381	55.79	0.457	0.478
FvF3H74	0.3564	0.297	0.25	0.3552	0.201	-0.007	0.401	56.15	0.459	0.487

FvF3H75	0.3394	0.2996	0.3053	0.358	0.203	-0.03	0.395	58.29	0.46	0.465
FvF3H76	0.3738	0.267	0.2591	0.3591	0.199	-0.068	0.372	58.87	0.461	0.489
FvF3H77	0.3958	0.2473	0.3546	0.278	0.183	-0.11	0.35	49.23	0.461	0.48
FvF3H78	0.2386	0.4318	0.2355	0.3202	0.218	0.066	0.453	53.55	0.465	0.458
FvF3H79	0.3925	0.317	0.3224	0.2743	0.208	-0.022	0.401	50.69	0.466	0.455
FvF3H80	0.1468	0.4642	0.1378	0.5019	0.224	0.11	0.473	48	0.466	0.473
FvF3H81	0.3623	0.3188	0.3892	0.2093	0.197	-0.07	0.373	52.08	0.469	0.463
FvF3H82	0.2447	0.4716	0.2265	0.3559	0.243	0.076	0.462	52.68	0.469	0.459
FvF3H83	0.3132	0.3843	0.3092	0.2675	0.2	-0.044	0.383	53.77	0.469	0.459
FvF3H84	0.5	0.212	0.3451	0.2311	0.238	-0.061	0.374	51.72	0.471	0.453
FvF3H85	0.3743	0.2696	0.2981	0.321	0.189	-0.046	0.392	56.88	0.473	0.457
FvF3H86	0.4519	0.251	0.3272	0.2563	0.212	-0.05	0.39	54.24	0.473	0.46
FvF3H87	0.3983	0.2554	0.3816	0.2371	0.185	-0.121	0.344	56.28	0.48	0.47
FvF3H88	0.4357	0.225	0.3583	0.2661	0.185	-0.139	0.331	48.97	0.482	0.454
FvF3H89	0.349	0.3255	0.3393	0.2464	0.193	-0.078	0.371	57	0.485	0.491
FvF3H90	0.3403	0.3681	0.3308	0.2489	0.211	-0.051	0.393	56.61	0.486	0.466
FvF3H91	0.3429	0.35	0.3532	0.2445	0.213	-0.076	0.381	58.19	0.486	0.463
FvF3H92	0.3227	0.3652	0.3529	0.2478	0.203	-0.051	0.393	57.65	0.486	0.469
FvF3H93	0.3227	0.3652	0.3529	0.2478	0.203	-0.051	0.393	57.65	0.487	0.463
FvF3H94	0.3147	0.3636	0.3676	0.2402	0.198	-0.068	0.383	56.64	0.492	0.485
FvF3H95	0.3603	0.3137	0.3423	0.2786	0.208	-0.055	0.389	55.82	0.493	0.447
FvF3H96	0.36	0.3455	0.3333	0.2456	0.192	-0.085	0.372	59.02	0.493	0.466
FvF3H97	0.2724	0.3586	0.3154	0.3465	0.211	-0.039	0.398	60.95	0.494	0.466
FvF3H98	0.2986	0.4173	0.2519	0.3061	0.247	0.068	0.46	55.1	0.496	0.479
FvF3H99	0.3213	0.3935	0.2519	0.302	0.25	0.049	0.448	56.57	0.497	0.459
FvF3H100	0.375	0.2578	0.3636	0.2909	0.19	-0.143	0.331	52.28	0.497	0.474

FvF3H101	0.3838	0.2362	0.3849	0.2863	0.218	-0.053	0.388	52.95	0.506	0.472
FvF3H102	0.3838	0.2362	0.3849	0.2863	0.218	-0.053	0.388	52.95	0.507	0.495
FvF3H103	0.44	0.1964	0.3258	0.3239	0.2	-0.114	0.342	47.83	0.507	0.478
FvF3H104	0.3879	0.3034	0.3343	0.2446	0.195	-0.025	0.394	56.9	0.512	0.469
FvF3H105	0.3287	0.3633	0.2473	0.3439	0.213	0	0.416	58.56	0.52	0.481
FvF3H106	0.3086	0.2037	0.354	0.4257	0.177	-0.189	0.3	57.57	0.529	0.479
FvF3H107	0.3696	0.2879	0.3514	0.2712	0.205	-0.022	0.398	52.41	0.535	0.481
FvF3H108	0.3772	0.2918	0.3442	0.2688	0.2	-0.024	0.396	51.66	0.539	0.485
FvF3H109	0.4034	0.2899	0.35	0.2732	0.226	-0.077	0.379	54.86	0.54	0.493
FvF3H110	0.3512	0.314	0.4017	0.2269	0.191	-0.045	0.391	54.74	0.54	0.51
FvF3H111	0.3299	0.354	0.341	0.2301	0.207	-0.017	0.402	57.68	0.549	0.504
FvF3H112	0.2136	0.4746	0.1741	0.3543	0.215	0.101	0.468	53.27	0.553	0.48
FvF3H113	0.2899	0.4454	0.2275	0.3607	0.264	0.004	0.427	55.24	0.559	0.491
FvF3H114	0.2787	0.4713	0.2566	0.2944	0.24	0.022	0.434	56.72	0.56	0.514
FvF3H115	0.3441	0.3151	0.3463	0.2594	0.197	-0.011	0.411	55.68	0.576	0.508
FvF3H116	0.375	0.2875	0.3576	0.2734	0.182	-0.103	0.348	52.51	0.579	0.49
FvF3H117	0.4615	0.2538	0.3421	0.2843	0.217	-0.148	0.348	50.92	0.58	0.489
FvF3H118	0.3497	0.3366	0.3636	0.2348	0.212	0.049	0.448	54.48	0.586	0.494
FvF3H119	0.3607	0.3464	0.2568	0.3125	0.257	0.082	0.466	48.08	0.595	0.491
FvF3H120	0.4318	0.25	0.3347	0.2844	0.218	-0.029	0.409	51.91	0.601	0.503
FvF3H121	0.3045	0.428	0.2743	0.2963	0.232	0.025	0.441	57.17	0.603	0.495
FvF3H122	0.2711	0.4437	0.2661	0.3022	0.235	0.108	0.478	54.98	0.609	0.526
FvF3H123	0.2534	0.4041	0.318	0.3253	0.205	0.039	0.447	53.06	0.626	0.512
FvF3H124	0.3471	0.2955	0.3247	0.311	0.2	-0.042	0.395	55.78	0.654	0.539
FvF3H125	0.3355	0.3094	0.3133	0.337	0.174	-0.037	0.391	58.07	0.676	0.584
FvF3H126	0.3545	0.3284	0.2642	0.3493	0.206	-0.074	0.372	61	0.766	0.597

Supplementary Table S4. Primer sequences of *FvF3H* gene family used for qRT-PCR

name	Forward primer(5'-3')	Reverse primer (5'-3')
FvF3H4	GGTGTCCAAGAGCCGATAAGGTG	TGTCCTGCAACAGAACAGTCATTCC
FvF3H6	TGGTCTCCTCATCCCTTCCTCAATC	TCTGTTTCTCTTCCACTGGCATAGC
FvF3H7	AGCAACTCGCTGAAGGTGAACTAC	CTGATTGAGGAAGGTGTGAGGAGAC
FvF3H10	AGGAAGGGATCGGCTGGATGTC	CGCACGGTGAATTGTAGTCTTGAAC
FvF3H12	TTGGCAACCGATGATGTCTTAGGC	CGTAGGCAGGCACTCCACAAG
FvF3H13	TCCACCATCCAAGAACAACACCATC	ACCTTCCATTACTCAGCACCTCCAG
FvF3H15	ATGTCCAGGCACTCTCATTGTCAAC	CTGCGACTGCTTCACCCTTTGG
FvF3H16	GAGAAGGAGGCATTGACGAAGGC	GTGTGGCGTTTGAGTCCGAGAG
FvF3H19	GAGTGTTGGACGAGATGCTGGAG	TCAGGCTCAGGACATGGAGGATAG
FvF3H23	TCGGGACTGGAAGGAGGTGTTTG	TGGAGATGGGCAAGGCGGATAG
FvF3H27	TGCGAGGACTTTGGCTTCTTCTATC	TAGTAGGACAGTGGTGCTCAGGATG
FvF3H30	TTAAGCGGTATCTGGAGGAGGTGAG	ATGGCGGATAGTGATTTGACAGCAG
FvF3H32	AGCCTGTCCCTAATGCCTTGGTAG	AAGGATGGATGAAGCTCGCAACTG
FvF3H34	AGTGACCAAAGTGCCAAAGTTCCTC	TGTTTCCGATGCTTCACGAATCTCC
FvF3H40	GCAGAGCAACTAATTCGCATCGTC	TCGGTTCGGGAGTGGAGTATGG
FvF3H44	CCGCACACTGACACCTCCATTG	ACCACCACACGATGAAGAACACTG
FvF3H53	CGGAGACACCAGCATTGTTCAAGAG	GGAGCCATTGCCATCCTCATCAC
FvF3H54	TGGCACAGGCAAACACTCAGATG	TTACAGATCCAGGCACAGGAACAAC
FvF3H58	TTACGCCTCATTCAGATGCCTCAG	ACGCTTCAGGAAGTGGCTTAACAG
FvF3H62	GCCGAGAGTTTAAGAGACCAAGACC	CCTGCATCGACGATCCCTTTCAC
FvF3H63	CCCGCAGCCTGAATTGACCATC	TGGAAGAAGCAACCCACAGAAACTC
FvF3H66	GAGTCTCTTGGGCTAAAGCGAAGTC	TTGTGGGCAAGCAGGATAGTAATGG
FvF3H67	CCAGCAGTCCAGTCATCTACAACAG	GCCCAAGAGCCTCAGACAACAAC
FvF3H69	TCCACAGCCAGAACTGACTCTAGG	CGGGCACAGGAGGTACATCAAAC
FvF3H70	GCGGCTTCCTCACTGTCCTTC	CCTGTGCTCTACACTCCTGAATCTG
FvF3H73	CAGGCGGTTTCAGTATCCCAGTG	TGACCTTCCTCACCATATCCCTACC
FvF3H79	CACTCGGACTGGCTGTTCATACTG	GCCCTTCACTCCCTCTGTTTGC
FvF3H80	GCGGCGTGGAACAAGGAGATC	GGCAGGGCGGGTAGTAGTGTC
FvF3H82	GCACTCACCTTCATCCTCCACAAC	TCTTCTCCTTGGGCGGCTCAC
FvF3H87	GCGAGTTCCATGAGCAGGATAGTG	AGCCGAGTGTGTCTCTCCAGTC
FvF3H88	GTCTGCGAGGACATGGTGAGTG	TTGTGGTGACTGGAGCAACTGTAAG
FvF3H89	CGCCTTGTCCACAGCCTGAAC	GCCATTTCCCATTGTGCTCAACTTG
FvF3H92	AGAAGAAGCCGAGAAGTAGCATTGG	TGAGGAGAGTGACTAGACCGTGATC
FvF3H94	ACTTCTTCAAGGTCCGAACACATCC	GGCTGAGGACAAGGAGGGTAGTAG
FvF3H97	CGGAGGAGATGAAGGTGGAGGAG	TGGTTGCGGACAGTAGGGATAGC
FvF3H103	CGGACACGAGCACCATAACCATAC	CACAACAAGAGCATCTGGAATTGGC
FvF3H104	AACTTCATCTTCTCGTGGTGCTTGG	ACTGGAGGACTCTTGACTGGTTCC
FvF3H110	ATGAGAGCGTGGGTATTGATGAAGC	TCGCAGCAGGTAGTTCGTTGAC
FvF3H112	GCCTCACCTTCTTCAACGACTCC	GCAACCACCCGCCGATATTCC
FvF3H120	TGCCAATGCCAGAATCACATCACTC	AGGCTTCAGAAAGAGAGGGCAAATC
FvF3H123	GAAGCCACCGAGGGAGTTGATTG	TGTTCTTGAAGCCAGTTCGTGAAGG

FvF3H126	GCACTTACCATTCTCCTCCAAGACC	CGGGCTAATCAGGGCATCATCC
FvGAPDH	CATTCATCACCACCGACTACA	GAAGGGTCTTCTCATCCTTGAC
